# Supplementary figures and images for: Membrane insertion of mitochondrial-encoded proteins regulates ribosome decoding speed
Source: Nat Struct Mol Biol. 2026 May 7;33(5):853–67. doi: 10.1038/s41594-026-01803-w (PMC13186706; doi:10.1038/s41594-026-01803-w)

Figure 1 b

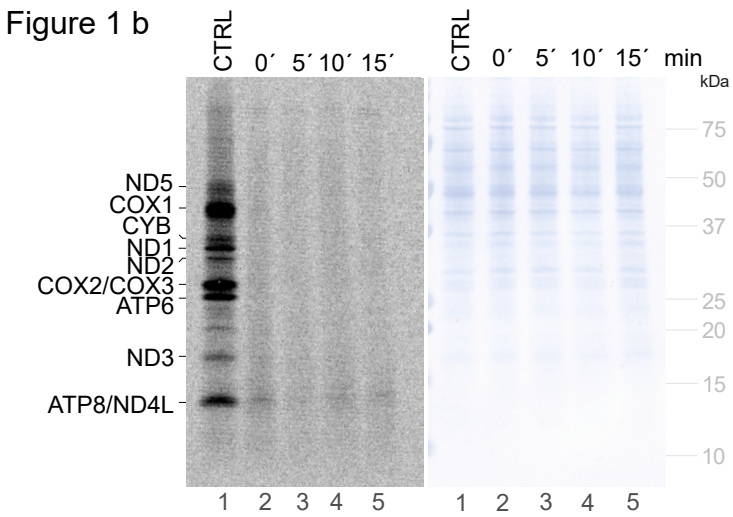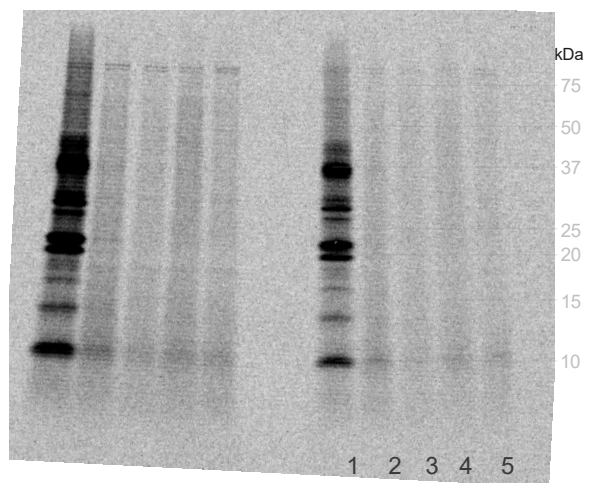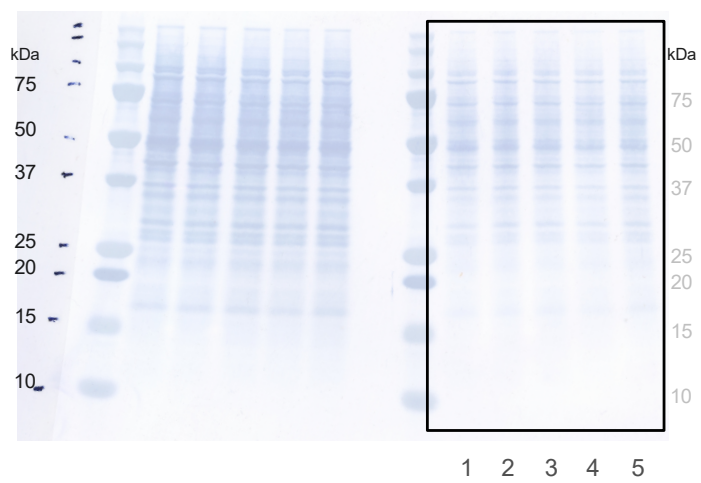

Raw data Figure 1 b Schoendorf et al., 2025

Figure 1 c

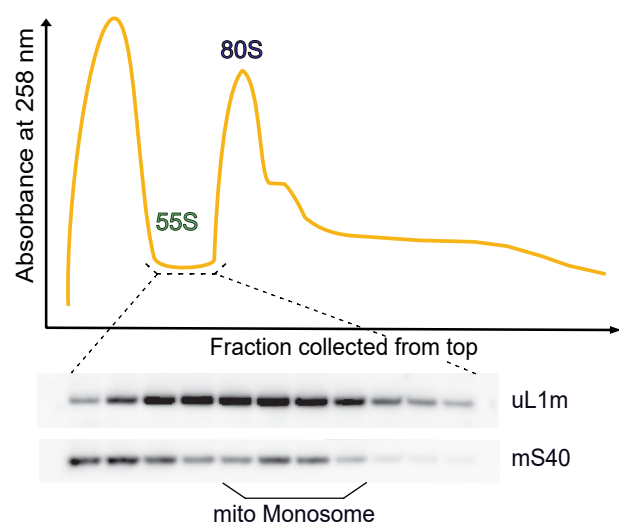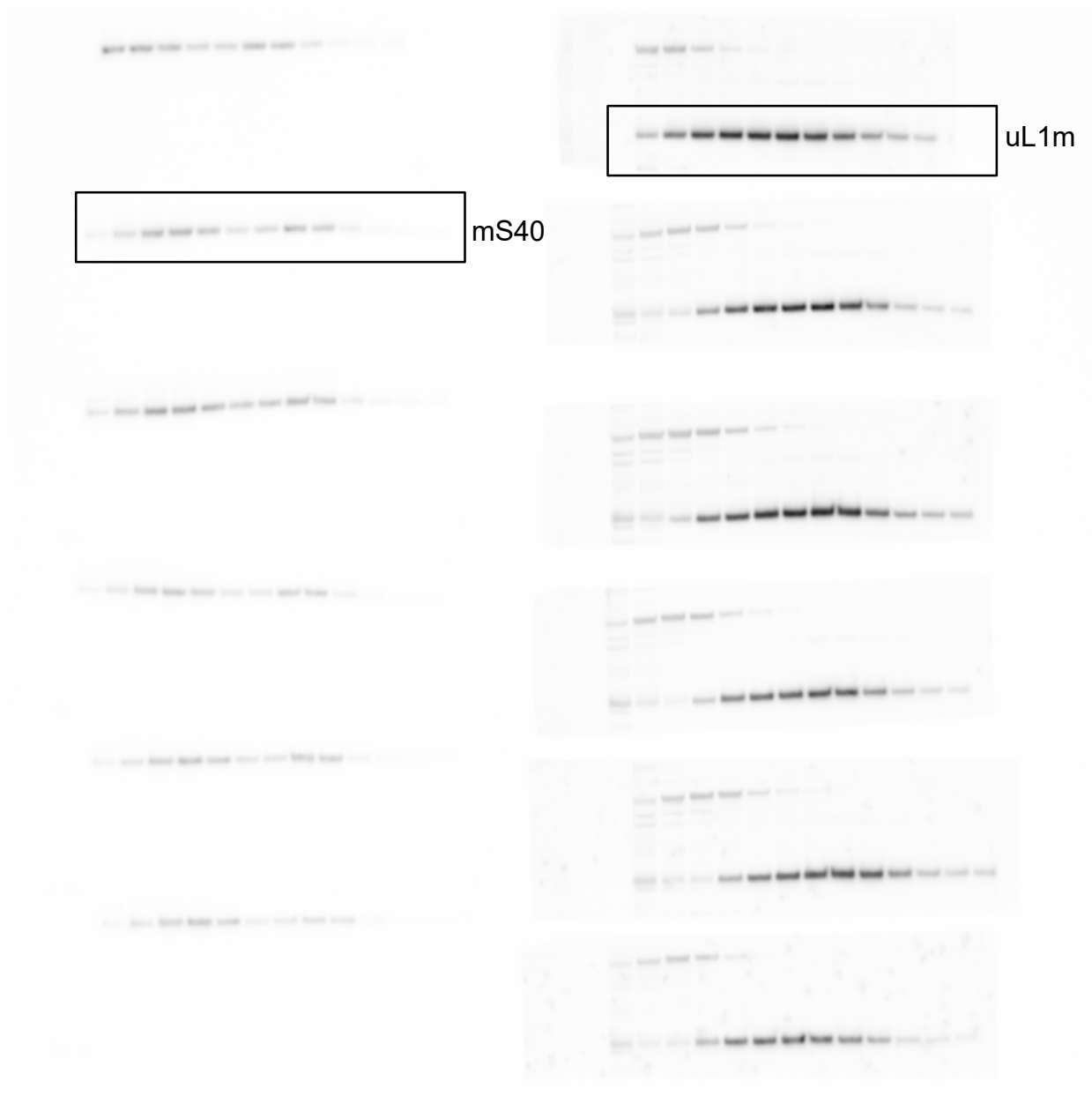

Figure 1 d

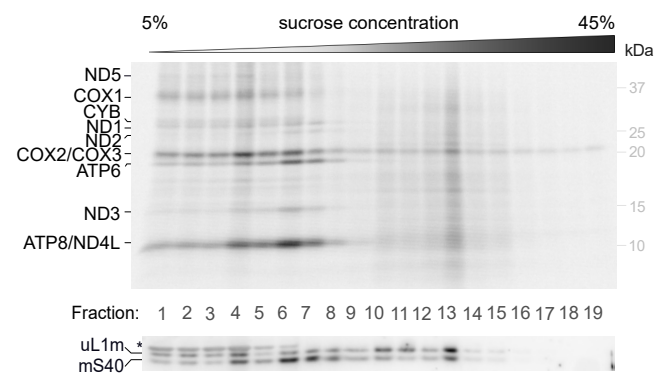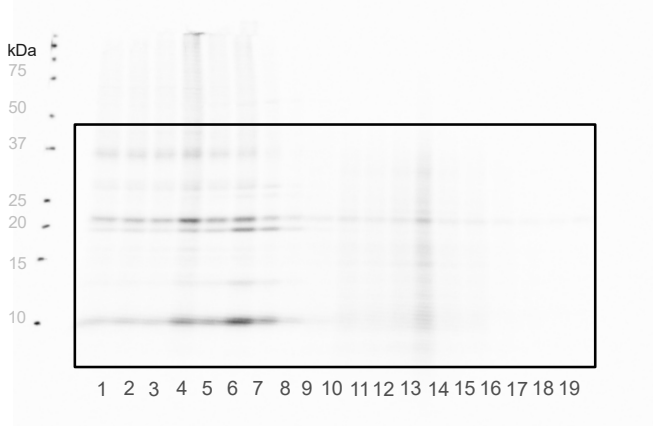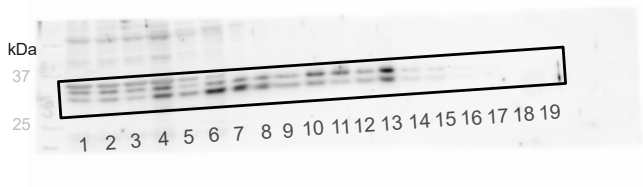

Raw data Figure 1 d Schoendorf et al., 2025

Supplement: Supplementary file 3 — Unprocessed western blots and/or gels. [file 41594_2026_1803_MOESM3_ESM.pdf]

Figure 2 b

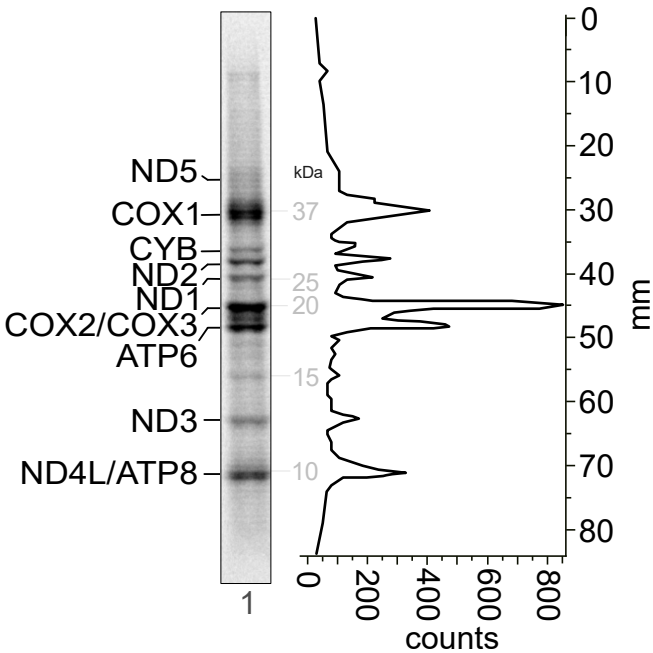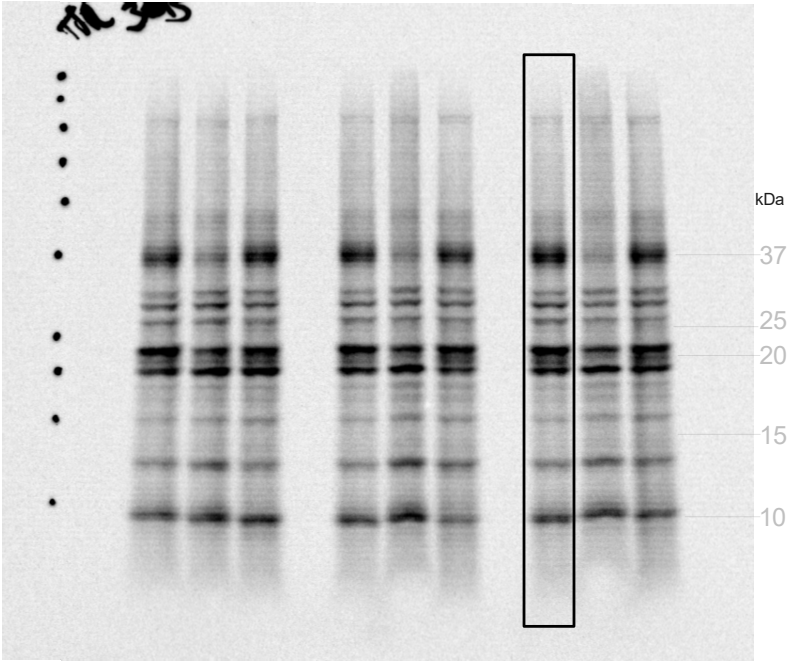

Raw data Figure 2 b Schoendorf et al., 2025

Supplement: Supplementary file 4 — Unprocessed western blots and/or gels. [file 41594_2026_1803_MOESM4_ESM.pdf]

Figure 6 b

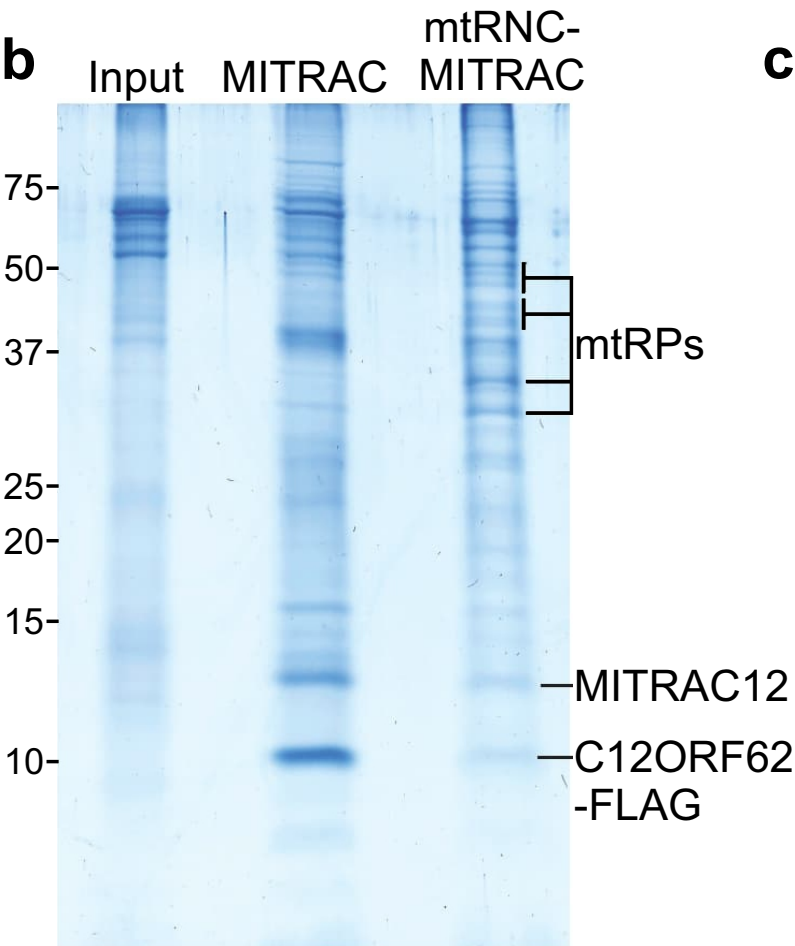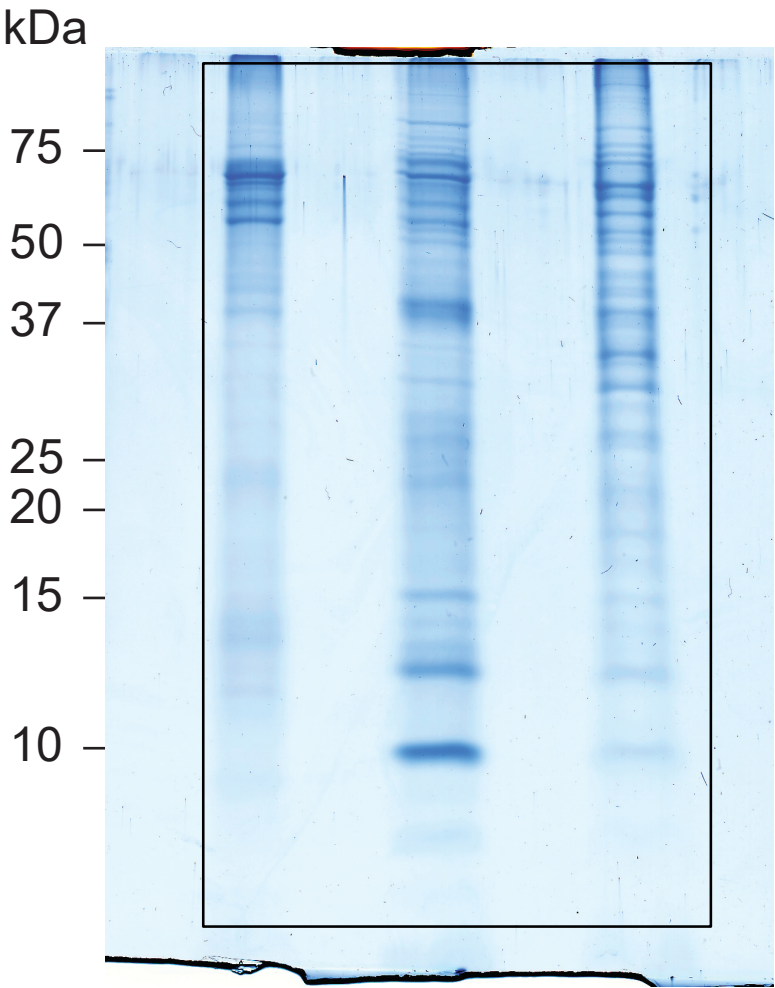

Supplement: Supplementary file 6 — Unprocessed western blots and/or gels. [file 41594_2026_1803_MOESM6_ESM.pdf]
