# Supplementary material for: Membrane insertion of mitochondrial-encoded proteins regulates ribosome decoding speed
Source: Nat Struct Mol Biol. 2026 May 7;33(5):853–67. doi: 10.1038/s41594-026-01803-w (PMC13186706; doi:10.1038/s41594-026-01803-w)
Supplement: Supplementary file 5 — Unprocessed western blots and/or gels. [file 41594_2026_1803_MOESM5_ESM.pdf]

Figure 4 a

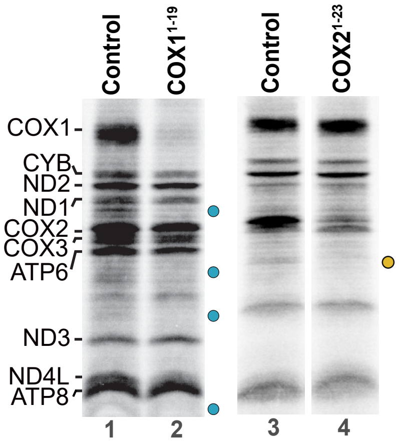

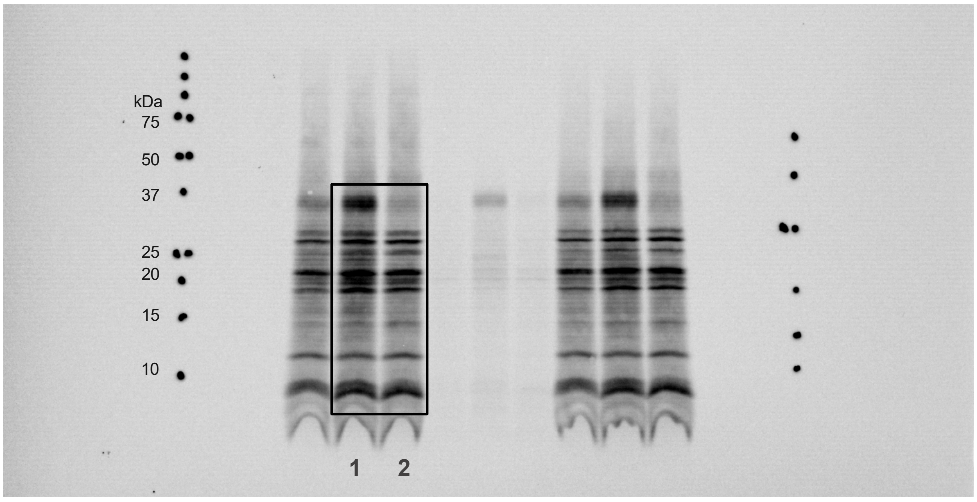

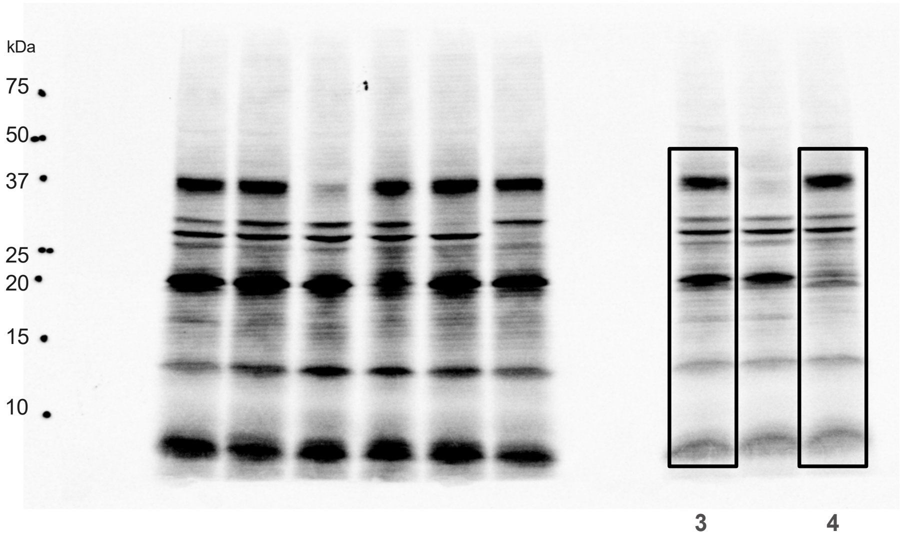

Raw data Figure 4 a Schoendorf et al., 2025

Figure 4 b

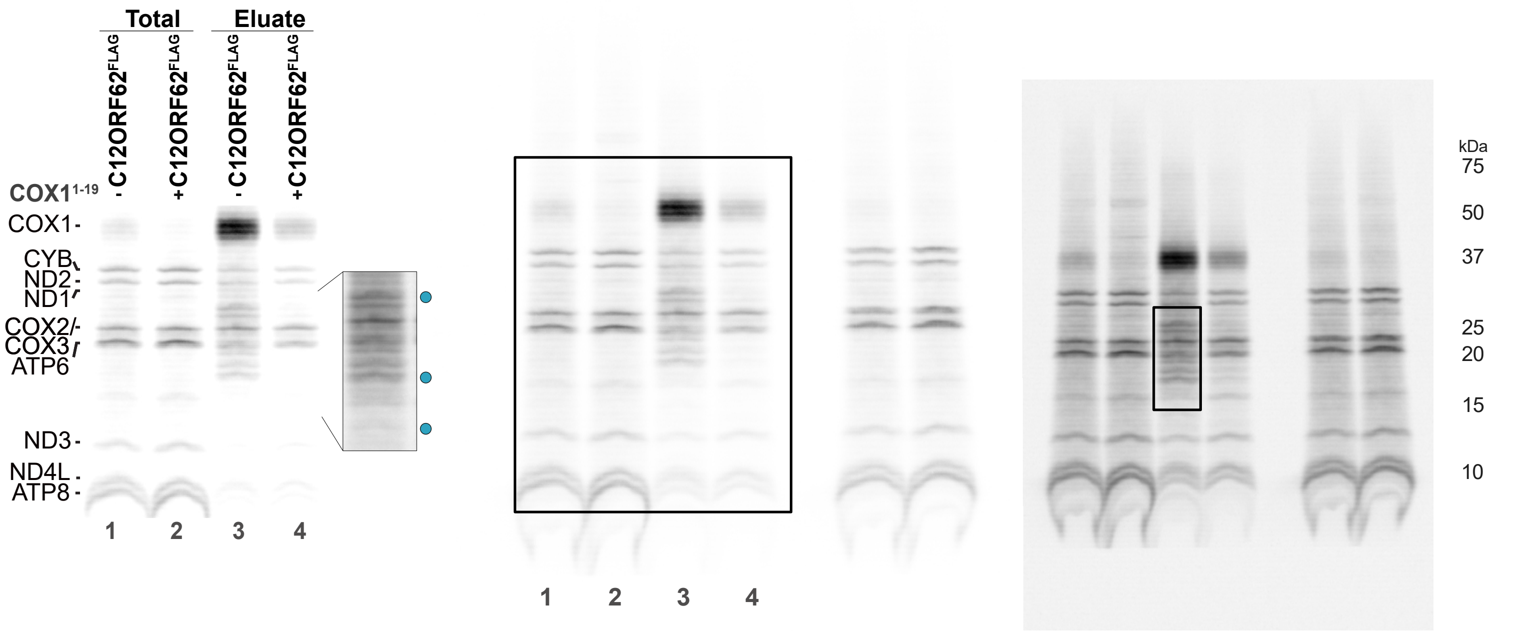

Raw data Figure 4 b Schoendorf et al., 2025

Figure 4 c

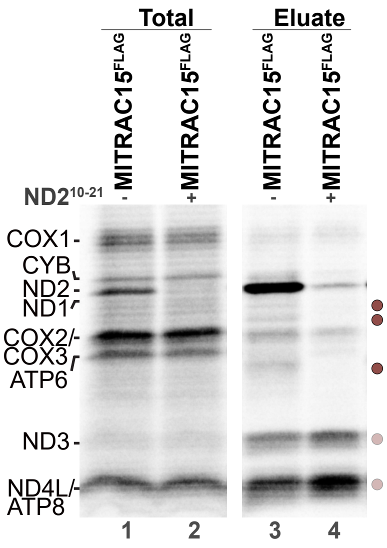

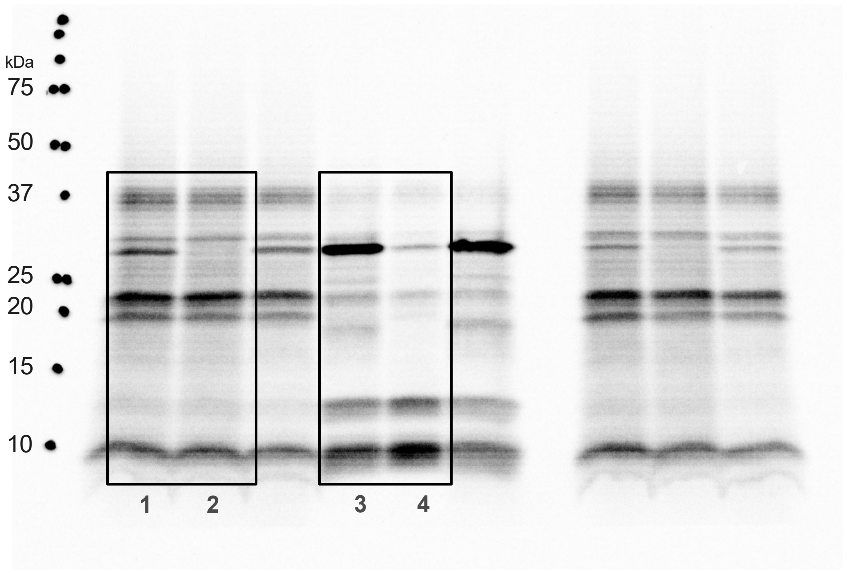

Raw data Figure 4 c Schoendorf et al., 2025
