# Supplementary material for: Membrane insertion of mitochondrial-encoded proteins regulates ribosome decoding speed
Source: Nat Struct Mol Biol. 2026 May 7;33(5):853–67. doi: 10.1038/s41594-026-01803-w (PMC13186706; doi:10.1038/s41594-026-01803-w)
Supplement: Supplementary file 7 — Unprocessed western blots and/or gels. [file 41594_2026_1803_MOESM7_ESM.pdf]

Extend data figure 1 a

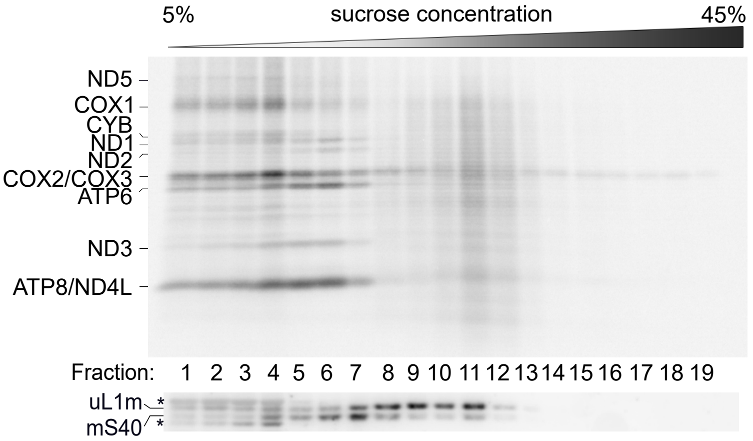

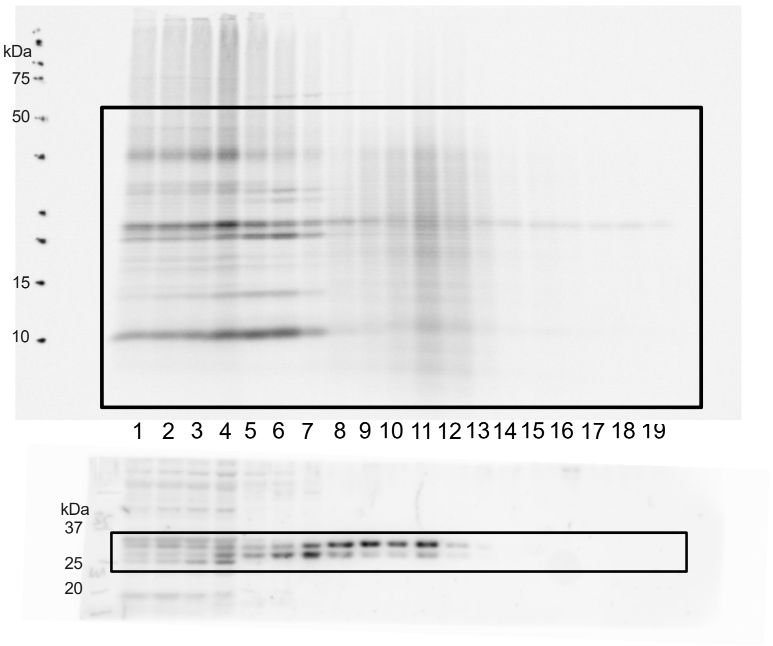

Rawdata extended data Figure 1 a Schoendorf et al., 2025

Extended data figure 1 c

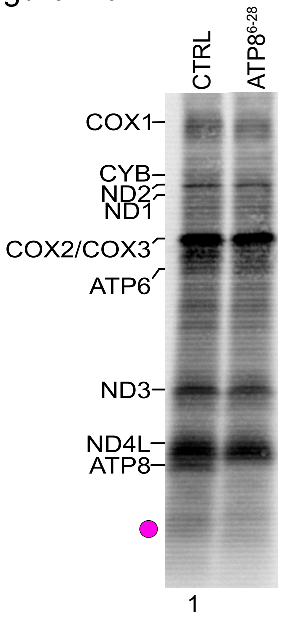

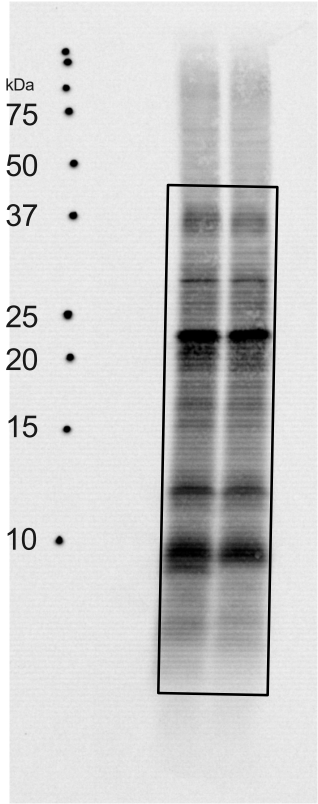

Rawdata extended data Figure 1 c Schoendorf et al., 2025
